# Dynamic Variations in Fecal Bacterial Community and Fermentation Profile of Holstein Steers in Response to Three Stepwise Density Diets

**DOI:** 10.3390/ani9080560

**Published:** 2019-08-15

**Authors:** Qinghua Qiu, Yangxiang Zhu, Xinjun Qiu, Chaoyu Gao, Jingjing Wang, Haibo Wang, Yang He, Muhammad Aziz ur Rahman, Binghai Cao, Huawei Su

**Affiliations:** 1 State Key Laboratory of Animal Nutrition, College of Animal Science and Technology, China Agricultural University, Beijing 100193, China; 2 Institute of Animal and Dairy Sciences, University of Agriculture, Faisalabad 35200, Pakistan

**Keywords:** fecal bacteria, dynamic variation, fermentation profile, dietary density

## Abstract

**Simple Summary:**

The gastrointestinal microbial ecosystem of cattle impacts their health and productivity. Collection of fecal samples provides a non-invasive and practicable way to explore the relationships between fecal microbiota and host productivity or health. Fecal bacteria are influenced by diet, feeding regime, animal age, and health status. However, dynamic variations in the fecal fermentation profile and microbiota composition of finishing steers in response to variable diets are limited. In the current study, we conducted an 11-month tracking investigation to uncover the dynamic variations in fecal fermentation profile and bacterial community in steers fed three stepwise density diets. We found that fecal bacterial diversity decreased as dietary density increased and as the fattening phase continued. Our results revealed that fecal organic acids and bacterial composition were influenced by diet and fattening period. Our results also indicated that time-dependent variations of fecal fermentation profile and microbiota composition exist in the long-term fattening of steers in addition to diet stimulation. This study will be beneficial to reducing fecal contamination from the origin by optimizing diet and fattening time.

**Abstract:**

The objective of this study was to track the dynamic variations in fecal bacterial composition and fermentation profile of finishing steers in response to three stepwise diets varied in energy and protein density. A total of 18 Holstein steers were divided into three groups in such a way that each group contained six animals and received one of three stepwise dietary treatments. Dietary treatments were C = standard energy and protein diet, H = high energy and protein diet, and L = low energy and protein diet. Animals were fattened for 11 months with a three-phase fattening strategy. Fecal samples were collected to evaluate the dynamics of fecal fermentation and bacterial composition in response to dietary treatments and fattening phases using 16S rRNA gene sequencing. Fecal acetate, propionate, and butyrate increased with increasing density of diet and as the fattening phase continued. The relative abundances of *Firmicutes* and *Bacteroidetes* dominated and showed 56.19% and 33.58%, respectively. Higher dietary density decreased the fecal bacterial diversity, *Firmicutes* to *Bacteroidetes* ratio, and the relative abundances of *Ruminococcaceae_UCG-005*, *Rikenellaceae_RC9_gut_group*, and *Bacteroides*, whereas higher dietary density increased the abundance of *Prevotella_9*. Our results indicated that both fecal fermentation profile and bacterial composition share a time-dependent variation in response to different dietary densities. This knowledge highlights that both diet and fattening phase impact fecal fermentation profile and bacterial composition, and may provide insight into strategies to reduce fecal contamination from the origin by optimizing diet and fattening time.

## 1. Introduction

In ruminants, the hindgut is regarded as a fermentation site with less buffering capacity when compared to the rumen [1]. The hindgut has similar rates of fermenting carbohydrates into volatile fatty acids (VFA) as the rumen, and it responds in parallel to the rumen to various substrates regarding the profiles of VFA [1,2]. However, sampling contents from the hindgut generally requires invasive operation or sacrificing of animals [3]. Doelman et al. (2019) reported that the concentration of isobutyric acid in feces could be indirect marker of fermentative characteristics for the hindgut. Therefore, some studies have recommended the collection of fecal samples as an alternative means to explore the relationship between the hindgut microbiota and host productivity [4,5].

Gastrointestinal microbial ecosystems have profound impacts on cattle health and productivity. Decline in fecal microbial diversity of pre-weaned dairy calves is related to a high incidence of diarrhea, and increased microbial diversity is associated with higher weight gain [6]. Recently, Lopes et al. (2019) found that fecal microbial populations are associated with feed efficiency. Previous reports have evidenced that feeding strategy or diet mainly influences bacterial composition but not archaeal community in fecal microbiota [7,8].

Many factors could impact composition of fecal bacteria, such as diet [8,9], feeding operation [10], animal age [11], and health status [6]. Diet plays a vital role in influencing fecal bacterial communities, and it has been studied extensively in recent years [7,12,13]. Zhang et al. (2018) explored the effect of a wide range of ratios of concentrate to forage on fecal community composition in heifers with a limit-feeding strategy. Plaizier et al. (2017) and Mao et al. (2012) reported that a high-grain diet induced subacute ruminal acidosis and greatly altered fecal bacterial community in cows. Additionally, age also impacts fecal bacterial community composition, and studies have focused on calves [6,11]. Klein-Jobstl et al. (2014) explored time-dependent variation of fecal microbial community before weaning in Simmental calves. Hua et al. (2017) [14] evaluated the variations in composition of ruminal microbiota in goats fed short or long-term high concentrate. However, data of dynamic alterations in fecal bacteria driven by various diets in finishing cattle are limited. Furthermore, there have been no reports investigating fecal bacterial variations in finishing cattle under different dietary densities as the fattening stage continues.

Generally, in beef cattle production systems, the fattening period consists of over six months and dietary density is normally increased continuously to reach optimum weight for slaughter [15,16]. The objective of this study was to investigate nutrition-driven changes in fecal fermentation profile and bacterial composition in finishing cattle during fattening phases.

## 2. Materials and Methods

### 2.1. Ethics Statement

The animal care and experimental procedures were handled strictly in accordance with the recommendations in the Guide for the Care and Use of Laboratory Animals of the National Institutes of Health of China. The protocols were approved by the China Agricultural University Animal Care and Use Committee (Permit No. AW09059102-2).

### 2.2. Animals, Experimental Design, and Sampling

A total of 18 Holstein steers, with body weight of 465 ± 38 kg and age of 413 ± 15 days, were selected from the same commercial beef cattle farm. Procured animals were reared on same diet, management, and climate conditions before start of the trial. Steers were randomly assigned to three treatments in such a way that each treatment had six animals. Experimental diets were standard energy and protein diet (C), high energy and protein diet (H, 110% of C), and low energy and protein diet (L, 90% of C). Diets were designed to meet or exceed the nutrition requirements of beef cattle (NRC) [17] and to maintain the same ratio of metabolizable energy (ME) to digestible protein in the intestines (DPI) (0.03 Mcal/g) to reduce potential effects due to protein balance [7]. Ingredients and nutrient composition of the diets are listed in Table 1. The trial was conducted from December 2017 to October 2018, and this time period was divided into three phases of 3, 3, and 5 months. The temperature and relative humidity were 1.46 °C and 34.53%, 21.12 °C and 55.68%, and 18.87 °C and 68.97% in phase 1 to phase 3, respectively. During the experimental period, animals were housed in individual pens and similar management practices were ensured for all experimental units. It was ensured that animals were well adapted to experimental feed during each phase of the trial. All steers had free access to feed and water. Animals were fed twice a day and feeding times were 7:30 and 16:30.

Fecal samples were collected before morning feeding for three consecutive days in each phase. Collected fecal samples were thoroughly mixed for individual steers to obtain representative samples. Obtained fecal samples were divided into two parts for genomic DNA extraction and fecal fermentation profile. Fecal samples obtained for genomic DNA extraction were immediately stored in liquid nitrogen and then transferred to a refrigerator at −80 °C. Fecal samples obtained for fecal fermentation profile were dissolved with quintuplicate sterile water and homogenized using a sterile homogenizer. The value of pH was immediately measured by a portable pH meter (Testo 205, Testo AG, Schwarzwald, Germany) in triplicate. The homogeneous mixture was centrifuged at speed of 6000 rpm/min for 30 min at 4 °C. Liquid supernatant obtained from previous step was stored at −80 °C, and VFA and ammonia nitrogen (NH_3_-N) were detected within two weeks. The concentration of NH_3_-N was determined according to the previously reported method [18] using a reader (Tecan, Mannedorf, Zurich, Switzerland).

### 2.3. VFA Detection and DNA Isolation

Primary centrifugal supernatants were thawed on ice after being removed from the −80 °C refrigerator. Centrifugation at 20,000× *g* for 20 min at 4 °C was performed to obtain supernatants, and the downstream measurements were based on these. A mixture of 200 μL metaphosphoric acid (250 g/L) and 800 μL fecal supernatants was used for subsequent detection of VFA, with 2-ethylbutyric acid (2.17 mL/L) as an internal standard. The concentration of VFA was detected by a gas chromatograph (GC-2014 Shimadzu Corporation, Kyoto, Japan), which was equipped with a 30 m capillary column (Rtx-Wax, 0.25 mm ID × 0.25 μm film, Restek, Evry, France). The injection volume of each sample was 0.4 μL and injector temperature was controlled at 220 °C. The oven program of the column was as follows: initial 110 °C for 30 s, up to 120 °C at 10 °C/min, 120 °C hold for 4 min, and continue to 150 °C at 10 °C/min, during which split ratio and flow rate were kept at 20:1 and 2.5 mL/min, respectively.

Bacterial DNA was extracted using OMEGA Stool DNA Kit (Omega Bio-Tek, Norcross, GA, USA) under the guidance of manual instructions, with the modification of a two-step bead-beating strategy, as described by Paz et al. (2016) [19]. Purity and quality of the genomic DNA were evaluated on 0.8% agarose gels, and the quantity of the DNA was detected by a NanoDrop spectrophotometer (NanoDrop 2000 Technologies Inc., Wilmington, DE, USA). In the current study, three samples were obtained from six steers by randomly mixing two steers’ fecal samples into one sample during phase 1, while five samples were randomly selected from six steers in both phase 2 and phase 3. Therefore, a total of 39 fecal samples were taken for DNA extraction.

### 2.4. 16S rRNA Gene Sequencing and Analysis

After preliminary evaluation of quality and quantity of extracted genomic DNA, a total of 39 DNA samples were delivered to Allwegene Company (Beijing) for PCR amplification and MiSeq sequencing. The primers and program of PCR reaction used were according to our previous study [15]. Briefly, the V3 to V4 hypervariable regions were amplified with a barcoded forward primer (5′-ACTCCTACGGGAGGCAGCAG-3′) and reverse primer (5′-GGACTACHVGGGTWTCTAAT-3′). A well-optimized 25 μL reaction system was constructed as follows: 12.5 μL of 2 × Taq PCR MasterMix, 3 μL of BSA (2 ng/μL), 2 μL of primer (5 μM), 2 μL of template DNA, and 5.5 μL of DNase/RNase-free deionized water. The PCR conditions were set as follows: initial incubation at 95 °C for 5 min; followed by 32 cycles of 95 °C for 45 s, 55 °C for 50 s, and 72 °C for 45 s; and a final extension step at 72 °C for 10 min.

The raw data were analyzed using the quantitative insights into microbial ecology (QIIME) version 1.8 pipeline (http://qiime.org/). Sequences were removed if they were shorter than 260 bp, chimeric or mismatched, had a quality score below 20, or had ambiguous bases. The filtered sequences were clustered into operational taxonomic units (OTUs) at a similarity level of 0.97 using the UPARSE pipeline (USEARCH Version 8.1.1861), to generate rarefaction curves and calculate richness and diversity indices. Taxonomic classifications for each OTU were obtained by assigning against the SILVA version 128 bacteria alignment database (http://www.arb-silva.de), which adopted the Ribosomal Database Project (RDP) classifier (http://sourceforge.net/projects/rdp-classifier/) with a confidence threshold of 70%. Herein, only relative abundances over 1% were reported.

In particular, the raw sequencing data involved in this study were submitted to Sequence Read Archive (SRA) of NCBI under the accession number PRJNA560144.

### 2.5. Statistical Analysis

For fecal pH value, NH_3_-N, and VFA, repeated measures in the generalized linear mixed model procedure of SPSS (version 20, IBM Corporation, Armonk, New York, United States) were taken. The model included fixed effects for the fattening phase and diet, and a random effect for the animal. The repeated effect was different fattening phases (n = 3). The generalized linear mixed model procedure of SPSS was selected to analyze differences among phases for a given genus, because animals were randomly selected from each phase in each group. In this model, the fixed effects were the fattening phase and diet and the random effect was the animal. Differences between the fattening phases and diets were compared using Tukey tests, and *p* < 0.05 was considered with significant difference, which has been marked with lowercase letters within the same row in tables.

## 3. Results

### 3.1. Fecal pH Value, VFA, and NH_3_-N Concentration

As shown in Table 2, the dietary treatment and fattening phase had significant influences on fecal pH value and VFA concentration, except for the effect of fattening phase on the concentration of isovalerate. The concentration of butyrate increased along with a decrease in pH values as dietary density increased. Significant increases in the concentrations of acetate, propionate, butyrate, and total volatile fatty acids were observed as fattening periods advanced.

### 3.2. Sequencing Depth and Coverage

A total of 3,720,787 clean tags were obtained after the rigorous quality control from 39 samples, with an average of 95,404 for each sample. Of the high-quality sequences, 99.96% were distributed between the lengths of 400 bp and 440 bp. OTUs across all samples were rarefied to the lowest sample depth (58,890 reads) based on the pseudo-random number generator of QIIME. The percentage of Good’s coverage indicated that the current sequencing depth could represent at least 99.2% of the bacterial community.

### 3.3. Alpha Diversity

Indices of alpha diversity here included Chao1, observed species, phylogenetic diversity (PD) whole tree, and Shannon index (Table 3). There was no interaction between diet and phase in the observed indices of alpha diversity. Steers fed C and L diets had higher Chao1, observed species, PD whole tree, and Shannon indices than that of steers fed H diet. Additionally, Chao1, observed species, PD whole tree, and Shannon indices decreased as fattening continued, with the above indices higher in phase 1 than that in phase 2 and phase 3.

### 3.4. Taxonomic Profiles

**Figure 1:**
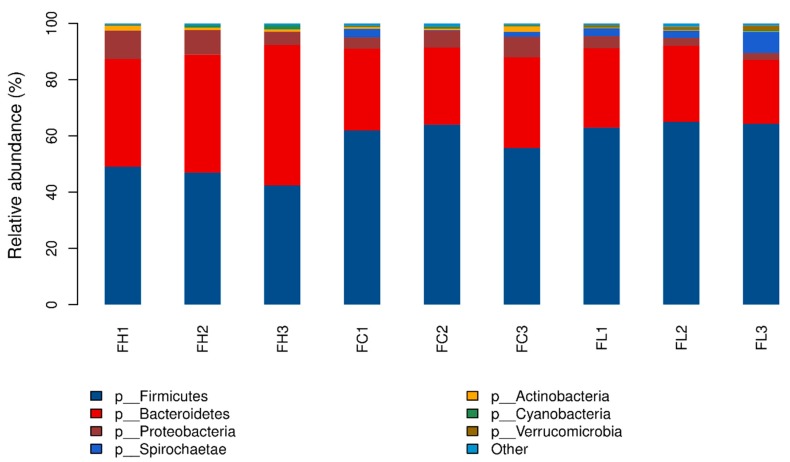
The relative abundance of fecal bacterial composition at the phylum level. FH, FC, and FL indicate stepwise high energy and protein diet group, standard energy and protein diet group, and low energy and protein diet group, respectively. Numbers 1–3 indicate phase 1, phase 2, and phase 3 of the fattening stage, respectively.

At the phylum level, *Firmicutes* was the most abundant of all groups (Figure 1), with *Firmicutes* accounting for 45.34%, 59.75%, and 63.47% in the H, C, and L groups, respectively. *Bacteroidetes* was the second dominant phylum, with abundance of 44.40%, 30.07%, and 26.26% in the H, C, and L groups, respectively. Other phyla with relative abundances greater than 3% were *Proteobacteria* in the three groups, and *Spirochaetae* in the L group. The ratio of *Firmicutes* to *Bacteroidetes* (F/B) ranged from 1.07 in the H group to 2.48 in the L group. Additionally, F/B was significantly higher in the C and L groups than in the H group (*p* < 0.001, data not shown).

At the genus level, there were 14, 24, and 22 genera with average abundances greater than 1% in the H, C, and L groups, respectively. Of these, *Prevotella_9*, *Ruminococcaceae_UCG-00*5, and *Ruminococcaceae_UCG-005* were the most abundant in the H, C, and L groups (Figure S1), with 21.04%, 12.55%, and 12.65%, respectively. An interaction between diet and phase was observed in *Prevotellaceae_UCG-003*. The relative abundances of *Prevotella_9*, *Succinivibrio*, *Prevotella_2*, *Alloprevotella*, and *Faecalibacterium* increased as dietary density increased, whereas *Ruminococcaceae_UCG-005*, *Rikenellaceae_RC9_gut_group*, and *Bacteroides* were higher in the feces of steers fed L diet (Table 4). *Ruminococcaceae_UCG-005* and *Bacteroides* decreased as the fattening phases continued, whereas *Phascolarctobacterium* increased as the fattening periods continued.

## 4. Discussion

### 4.1. Fecal Fermentation Profile

Bacteria, protozoa, and fungi are present in the hindgut of cattle, and they are responsible for the fermentation of undigested nutrients, resulting in the production of VFA and NH_3_-N [1,9]. Fresh feces could be a suitable alternative to hindgut samples, and feces is easy and practicable to obtain repeatedly [9]. In the present study, we took measurements of fecal VFA to evaluate variations in fermentation patterns of the hindgut, because it is a noninvasive and friendly method for the steers and fecal VFA could provide indirect markers of fermentative parameters for the hindgut [3,4].

The pH values of feces detected in the H group were low, as compared to findings of previous researchers who reported the range of pH between 6.05 to 7.15 [9,20,21]. These differences could be explained by composition of the diets and the dilution ratio of the feces. Li et al. (2012) reported a fecal pH of 6.57 when starch content was 21.3% in the diet of dairy cows and feces were diluted with half-weight distilled water. Mao et al. (2012) observed a fecal pH value of 7.15 in dairy cows fed 40% concentrate on a dry matter (DM) basis, and a fecal pH value of 6.42 in diet with 70% concentrate on DM basis, when feces were diluted with equal amount of water. Calamari et al. (2018) reported a fecal pH value of 6.05 in cows fed diets with a starch content of 25.1% on a DM basis. In this study, a low fecal pH value was observed in the H group, which was due to higher starch contents in the H group as compared to all above studies. Similarly, the lower pH in H group can also be justified by the higher starch contents of the H diet as compared to the C and L diets. The low values of pH (from 5.70 to 5.00) as fattening phases continued in the H group indicated that hindgut acidosis occurred at the last phase in the current study, as described in a previous study stating that a fecal pH < 5.00 is indicative of hindgut acidosis [1].

The NH_3_-N concentration in feces is affected by the efficiency of the microbial population and the supply of energy in the colon [22]. Previous studies have reported that higher fecal NH_3_-N represents a deficiency of available energy to assimilate ammonia into microbial protein [23]. In the current study, we observed no significant differences in NH_3_-N concentration in feces due to dietary treatment and fattening phase, which could be partly explained by the fact that the diets had similar ruminal degradable protein balance. However, in the current study, the value of NH_3_-N was lower than in the report of Sato and Nakajima (2005), who reported a concentration of 4.3 mmol/L in a 4× dilution of fresh feces, and the difference of NH_3_-N concentration between the current study and the study of Sato and Nakajima (2005) may be due to the different dilution ratios and diet compositions.

Previous studies have reported that fecal total VFA was influenced by production of VFA in the hindgut, and the amount of carbohydrates flowing into the hindgut determined the production of VFA [1,20]. Therefore, it is obvious that measurement of fecal VFA provides an indirect presentation of the fermentation pattern occurring in the hindgut [20]. In this study, the concentrations of total VFA were similar to the findings of Mao et al. (2012) and VFA concentration was increased linearly by increasing the density of the diet and with advancing fattening stage, except for phase 1, in which the L group had a concentration of total VFA below 60 mM. The exception might be partly explained by the fact that the low starch content in phase 1 of the L group reduced the production of VFA in the hindgut. Similarly to the report of Zhang et al. (2018), propionate concentration in feces progressively increased as the dietary density increased and fattening phase continued. The fecal acetate to propionate ratio (A/P) in this study showed a phase-progressive reduction, which is in agreement with the fact that the A/P decreased as ratio of concentrate to forage increased [24]. In addition, an A/P below 2.2 is considered an indicator of unhealthy animals and impaired performance in cows [15,24]; in the current study, the A/P in phase 3 of the H group was 1.82, which indicates the diet had negative effects on the steers’ health and may influence production performance. Furthermore, the lower A/P in phase 3 indicates that the animals fed the H diet underwent hindgut acidosis. The average butyrate concentration in the H group was 13.5 mM, which is higher than 8 mM, a threshold value for interruption of the intestinal barrier and which might induce intestinal epithelial cell apoptosis [25].

### 4.2. Fecal Bacterial Diversity and Profile

Chao1 values and observed species evaluate the richness of a community, while PD whole tree and Shannon indices assess the community from the point of view of diversity [7,12]. Plaizier et al. (2017) reported that dietary stimulation had similar effects on rumen and fecal bacterial diversity. However, Azad et al. (2019) found that microbiota diversity of rumen and feces responded differently to dietary treatment. In this study, bacterial richness and diversity of feces decreased as dietary density increased and as the fattening phase continued, which is consistent with numerous reports in which high energy or proportion of concentrate decreased ruminal and fecal alpha diversity [12,14]. These findings also support the report of Plaizier et al. (2017), as described before.

*Firmicutes* and *Bacteroidetes* represented approximately 90% of the bacterial community, specifically 89.74%, 89.82%, and 89.73% in the H, C, and L groups, respectively. Previous evidence reported that the F/B was associated with energy utilization and fat deposition in mice [26]. A recent study in bulls revealed that higher F/B was related to lower body weight and less intramuscular fat content [15]. Our results from fecal samples showed a similar correlation between dietary energy and F/B to that of Wang et al. (2019), who reported that increased dietary energy resulted in decreased F/B of ruminal samples. *Proteobacteria*, previously not deemed to be a major phylum, plays a great role in the formation of biofilms and digestion of soluble carbohydrates [15,27,28]. In this study, the relative abundance of *Proteobacteria* varied from 2.19 to 10.21%, which was higher than in a previous report [7], and we speculated that it was the diets that resulted in the difference. Additionally, *Proteobacteria* was generally high in the H group, which is in accordance with the fact that *Proteobacteria* is actively involved in digesting soluble carbohydrates [27], such as the high starch content in the H group. *Spirochaetae*, a phylum with a relative abundance of 4.54% detected in the L group, are possibly involved in degrading fiber materials [28]. *Treponema*, a genus belonging to *Spirochaetae*, is responsible for scavenging products of cellulose breakdown [29]. Our data indicated that the high fiber diet harbored greater proportions of *Spirochaetae* and *Treponema*, i.e., higher abundances than in the L and C groups, which is consistent with previous reports [7,12].

Variations at the genus level provided a deep insight into the profile of bacterial community composition in response to dietary density and fattening phase. *Ruminococcus* and *Prevotella* have previously been regarded as the most abundant genera of *Firmicutes* and *Bacteroidetes* in rumen, respectively [12]. However, the dominant genera in feces varied with diets, e.g., *Faecalibacterium* and *Prevotella* in the H group, *Ruminococcus* and *Prevotella* in the C group, *Ruminococcus* and *Rikenellaceae_RC9_gut_group* in the L group. These differences between rumen and lower tract could be interpreted as being due to the available nutrients for fecal microbiota differing from those of rumen microbes in both types and amounts [7].

*Ruminococcaceae_UCG-005* is a genus of the family *Ruminococcaceae*, which plays a pivotal role in digesting fiber and is more abundant in forage-fed animals [7,30]. *Ruminococcaceae_UCG-005* consistently decreased as dietary density increased, as well as with progression of the fattening phase, which was due to the lower fiber content in the higher density diet. *Prevotella_9*, belonging to the family *Prevotellaceae*, contributes greatly to starch degradation and is involved in proteolysis [31]. In this study, steers fed the H diet were significantly higher in relative abundance of *Prevotella_9* due to the higher starch content in the H diet than that of the C and L diets.

*Bacteroides* was previously believed to be the second largest genus in *Bacteroidetes*, represented by 27 species in feces [32]. *Bacteroides* was widely detected in all cattle raised on various diets, and *Bacteroides* is considered more abundant in cattle fed a diet rich in grain [8]. However, a meta-analysis on cattle feces revealed that it may play a role in degrading undigested fibers in the rumen [13]. In this study, the average relative abundance of *Bacteroides* was 1.00%, 3.87%, and 6.19% in the H, C, and L groups, respectively. In addition, the relative abundance of *Bacteroides* increased with diets of higher fiber content and decreased as the fattening phases continued (where lower fiber content diet was given), indicating that *Bacteroides* may be involved in fiber digestion in feces. However, Zhang et al. (2018) found no significant differences in the relative abundance of *Bacteroides* between heifers fed with a wide ratio of dietary forage to concentrate, implying that more investigations on diets and the ecological balances of certain species should be conducted to uncover the functions of *Bacteroides*.

*Succinivibrio* was the predominant genus of *Proteobacteria* in feces [32], with an average abundance of 4.93% in the three groups. *Succinivibrio dextrinosolvens*, a genus belonging to *Succinivibrio*, plays a vital role in fermenting various nonstructural carbohydrates into succinate and formate, and has been found to be the predominant genus in transitioning to high-grain diet [33,34]. In this study, a higher relative abundance of *Succinivibrio* was found in the feces of steers fed the H diet than that of steers fed the L diet, whereas no differences were observed between steers fed C and L or H and C; perhaps an insufficient increase of starch content could explain this. *Rikenellaceae _RC9_gut_group*, a genus belonging to the family *Rikenellaceae*, accounted for on average 4.54% of the total bacteria. Little is known about metabolic function of this family, and a previous researcher has speculated that it may be involved in the primary or secondary degradation of carbohydrates [35]. In the present study, the relative abundance of *Rikenellaceae _RC9_gut_group* increased as dietary fiber content increased, and a lower trend was also observed as fattening phases advanced; these findings may provide evidence for an alternative metabolic function of fiber degradation for this genus. The relative abundance of *Faecalibacterium* was 5.57% in the H group, whereas low abundances (0.68% and 0.03%) were detected in the C and L groups, respectively. These observations are supported by previous studies, where *Faecalibacterium* could be commonly detected in the feces of cattle fed a diet rich in grain, but rarely in forage-based diet [8,32]. In addition, the higher concentration of butyrate in the H group could be well explained by a higher relative abundance of *Faecalibacterium*, because *Faecalibacterium* greatly contributes to butyrate formation in the hindgut [13,36].

## 5. Conclusions

This study was the first to track the dynamic variations in fecal bacterial community and fermentation profile of finishing steers in response to various stepwise density diets. Our results showed that fecal VFA increased while pH value decreased when the high-density diet was fed to steers. The steers fed the stepwise high-density diet showed threatened health, as seen by continuously low fecal pH and high butyrate concentration. *Firmicutes* and *Bacteroidetes* predominated the relative abundances at the phylum level, and the ratio of *Firmicutes* to *Bacteroidetes* decreased as dietary density increased. The high-density diet generally displayed higher relative abundance of *Prevotella_9*, *Succinivibrio*, *Prevotella_2*, *Alloprevotella*, and *Faecalibacterium*, whereas the low-density diet displayed higher relative abundance of *Ruminococcaceae_UCG-005*, *Rikenellaceae_RC9_gut_group*, and *Bacteroides*. These findings suggest that *Bacteroides* and *Rikenellaceae _RC9_gut_group* may be involved in fiber degradation in the hindgut. This study may provide insight into reducing fecal contamination from the origin by optimizing diet and fattening time. Expanding knowledge of the relationships among the bacterial compositions of feces, hindgut, and rumen will simplify the sampling methods used to explore associations between microbiota and productivity.

## Figures and Tables

**Table 1:** Ingredients and Nutrient Composition of the Experimental Diets.

Item ^1^	Phase 1			Phase 2			Phase 3		
	H	C	L	H	C	L	H	C	L
Ingredients, % of DM									
Corn	52.94	41.44	29.94	58.75	46.77	34.79	61.99	50.08	38.17
Wheat	7.32	5.73	4.14	7.17	5.71	4.25	8.78	7.09	5.40
Soybean meal	9.41	7.37	5.32	9.37	7.46	5.55	8.97	7.25	5.53
*Leymus chinensis*	28.18	43.78	59.38	21.59	37.58	53.57	16.94	32.90	48.86
Calcium carbonate	0.72	0.56	0.41	0.78	0.62	0.46	0.83	0.67	0.51
Sodium bicarbonate	0.00	0.00	0.00	0.78	0.62	0.46	0.83	0.67	0.51
Sodium chloride	0.71	0.56	0.40	0.78	0.62	0.46	0.83	0.67	0.51
Vitamin–mineral premix ^2^	0.72	0.56	0.41	0.78	0.62	0.46	0.83	0.67	0.51
Nutrient composition, ^3^ % of DM									
ME ^4^, Mcal/kg	2.71	2.53	2.35	2.77	2.58	2.40	2.82	2.64	2.46
DPI ^5^	9.41	8.32	7.23	9.49	8.45	7.41	9.54	8.55	7.56
RDPB ^6^	0.30	0.30	0.29	0.30	0.29	0.28	0.30	0.29	0.28
NDF	27.7	37.1	46.4	23.6	33.2	42.8	20.8	30.4	40.0
ADF	14.6	20.2	25.8	12.1	17.9	23.7	10.4	16.2	21.9
Crude fat	3.46	3.25	3.05	3.56	3.34	3.13	3.61	3.40	3.19
Starch	43.7	34.8	25.9	47.6	38.5	29.3	50.8	41.6	32.3
NFC ^7^	52.0	43.7	35.4	54.9	46.7	38.6	60.3	51.6	42.9
ME/DPI, Mcal/g	0.03	0.03	0.03	0.03	0.03	0.03	0.03	0.03	0.03
Calcium	0.47	0.48	0.50	0.46	0.48	0.49	0.45	0.47	0.49
Phosphorus	0.29	0.26	0.23	0.29	0.27	0.24	0.30	0.27	0.24

^1^ H = stepwise high energy and protein diet group; C = stepwise standard energy and protein diet group; L = stepwise low energy and protein diet group. ^2^ Manufactured by Tangshan Mahanen Feed Co., Ltd., Tangshan, China; premix provided the following per kg of dry matter (DM): 5000 IU of vitamin A, 3000 IU of vitamin D3, 45 mg of vitamin E, 60 mg of Fe, 63 mg of Zn, 99 mg of Mn, 200 mg of Cu, 0.5 mg of Se, 1.1 mg of I, 0.45 mg of Co, 877.4 g of rice bran. ^3^ DM, dry matter; TDN, total digestible nutrients; ME, metabolizable energy; NDF, neutral detergent fiber; ADF, acid detergent fiber; NFC, non-fiber carbohydrates. ^4^ Estimated as ME = total digestible nutrients × 0.04409 × 0.82. ^5^ DPI, digestible protein in the intestines. ^6^ RDPB, ruminal degradable protein balance.^7^ Estimated as NFC = 100 − (NDF + CP + ether extract + ash).

**Table 2:** Fecal pH Value, Volatile Fatty Acid and Ammonia Nitrogen Concentration of Holstein Steers Fed with Three Stepwise Density Diets.

Item ^1^		H ^2^			C ^3^			L ^4^		SEM ^5^	*p*-value ^6^		
	HP1	HP2	HP3	CP1	CP2	CP3	LP1	LP2	LP3		Diet	Phase	D × P
pH value	5.70 ^c^	5.30 ^d^	5.00 ^d^	6.26 ^b^	6.19 ^b^	6.22 ^b^	6.82 ^a^	6.83 ^a^	6.68 ^a^	0.079	<0.001	<0.001	0.001
Acetate, mM	41.2 ^bc^	48.3 ^ab^	50.0 ^a^	42.5 ^bc^	43.1 ^abc^	44.7 ^abc^	30.8 ^d^	39.1 ^c^	45.6 ^abc^	1.599	<0.001	<0.001	0.005
Propionate, mM	13.8 ^bc^	17.2 ^b^	25.7 ^a^	13.6 ^bc^	15.0 ^bc^	17.1 ^b^	11.8 ^c^	14.1 ^bc^	14.9 ^bc^	0.973	<0.001	<0.001	<0.001
Isobutyrate, mM	1.10 ^bcd^	0.89 ^d^	1.44 ^ab^	1.16 ^bcd^	1.28 ^bc^	1.70 ^a^	0.97 ^bcd^	0.93 ^d^	1.70 ^a^	0.068	<0.001	<0.001	0.025
Butyrate, mM	8.31 ^c^	12.0 ^b^	20.2 ^a^	7.27 ^c^	6.74 ^cd^	8.71 ^c^	4.49 ^d^	6.89 ^cd^	7.42 ^c^	0.567	<0.001	<0.001	<0.001
Isovalerate, mM	0.24 ^ab^	0.25 ^ab^	0.12 ^ab^	0.22 ^ab^	0.18 ^ab^	0.27 ^a^	0.08 ^b^	0.07 ^b^	0.17 ^ab^	0.041	0.003	0.841	0.015
Valerate, mM	0.08 ^b^	0.43 ^a^	0.46 ^a^	0.48 ^a^	0.49 ^a^	0.53 ^a^	0.48 ^a^	0.55 ^a^	0.54 ^a^	0.033	<0.001	<0.001	<0.001
TVFA, mM	65.8 ^c^	78.4 ^b^	96.4 ^a^	65.3 ^c^	66.7 ^c^	72.9 ^bc^	48.6 ^d^	63.2 ^c^	70.1 ^bc^	2.255	<0.001	<0.001	<0.001
BCVFA, mM	1.15	1.11	1.56	1.34	1.40	2.02	1.02	1.04	1.75	0.097	<0.001	<0.001	0.460
A/P	2.96 ^a^	2.88 ^a^	1.82 ^b^	3.12 ^a^	2.91 ^a^	2.65 ^b^	2.60 ^a^	2.72 ^a^	2.77a	0.123	0.005	<0.001	<0.001
NH_3_-N, mg/dL	3.18	2.92	2.42	2.38	2.52	2.92	2.54	2.71	2.52	0.422	0.724	0.955	0.633

^1^ TVFA: total volatile fatty acid; BCVFA: branched-chain volatile fatty acid, equal to isobutyrate and isovalerate; A/P: ratio of acetate to propionate; NH_3_-N: ammonia nitrogen. ^2^ H = stepwise high energy and protein diet, where HP1 indicates phase 1 in H group, HP2 indicates phase 2 in H group and the similar pattern for HP3 and C and L groups. ^3^ C = stepwise standard energy and protein diet, where CP1 indicates phase 1 in C group, and the similar pattern for CP2 and CP3. ^4^ L = stepwise low energy and protein diet, where LP1 indicates phase 1 in L group, and the similar pattern for LP2 and LP3. ^5^ SEM = standard error of means. ^6^ Diet = the effect of diets, Phase = the effect of phases, D × P = the interaction between diet and phase, lowercase letters are marked only when the interaction was significant. ^a–d^ values within the same row with different superscripts differ.

**Table 3:** Fecal Alpha Diversity Indices of Holstein Steers Fed with Three Stepwise Density Diets.

Item ^1^		P1			P2			P3		SEM ^2^	*p*-value ^3^		
	H	C	L	H	C	L	H	C	L		Diet	Phase	D × P
Chao1	925.6	1212.4	1180.9	654.8	1008.3	1002.5	637.2	985.7	890.5	59.3	<0.001	<0.001	0.854
Observed species	674.3	950.6	925.0	477.6	750.0	802.4	466.8	722.4	731.9	44.6	<0.001	<0.001	0.891
PD whole tree	52.75	67.33	67.54	42.68	58.80	60.51	43.38	57.57	54.45	2.62	<0.001	<0.000	0.724
Shannon index	6.31	7.30	7.34	5.87	7.08	7.20	5.81	6.83	7.07	0.16	<0.001	0.024	0.880

^1^ P1 indicates phase 1, P2 indicates phase 2, P3 indicates phase 3; H = stepwise high energy and protein diet, C = stepwise standard energy and protein diet, L = stepwise low energy and protein diet. ^2^ SEM = standard error of means. ^3^ Diet = the effect of diets, Phase = the effect of phases, D × P = the interaction between diet and phase, lowercase letters are marked only when the interaction was significant.

**Table 4:** Fecal Genus (Relative Abundance >1%) Composition of Holstein Steers Fed with Three Stepwise Density Diets.

Item ^1^		P1			P2			P3		SEM ^2^	*p*-value ^3^		
	H	C	L	H	C	L	H	C	L		Diet	Phase	D × P
*Ruminococcaceae_UCG-005*	7.66	14.52	16.69	4.77	13.83	13.50	2.09	11.13	9.76	0.976	0.001	<0.001	0.509
*Prevotella_9*	20.35	1.21	0.44	21.41	3.53	0.26	21.71	9.71	0.26	7.929	<0.001	0.407	0.592
*Succinivibrio*	9.48	3.85	4.41	7.68	4.87	2.94	4.17	5.25	2.34	1.408	0.008	0.249	0.248
*Rikenellaceae_RC9_gut_group*	0.84	5.80	8.37	1.03	5.36	8.42	1.55	2.88	6.38	0.841	<0.001	0.079	0.225
*Bacteroides*	1.25	5.38	8.46	1.51	4.70	6.16	0.35	1.49	3.55	0.848	<0.001	<0.001	0.229
*Clostridium_sensu_stricto_1*	2.96	2.53	2.57	3.36	2.70	2.17	3.47	5.58	3.64	0.879	0.537	0.060	0.471
*Ruminococcaceae_UCG-014*	2.43	2.14	1.99	2.67	2.99	4.61	3.02	2.21	1.78	0.698	0.819	0.067	0.182
*Phascolarctobacterium*	2.29	1.54	1.43	3.63	3.29	2.26	3.18	2.95	2.33	0.604	0.134	0.043	0.952
*Prevotella_2*	7.05	0.23	0.00	6.12	0.53	0.00	6.61	2.34	0.01	1.007	<0.001	0.598	0.722
*Alloprevotella*	1.89	1.90	0.25	2.65	1.67	1.49	5.26	2.37	1.61	0.875	0.020	0.070	0.433
*Faecalibacterium*	4.49	0.19	0.01	5.47	0.70	0.05	6.62	0.96	0.01	0.943	<0.001	0.493	0.874
*Prevotellaceae_UCG-003*	0.28 ^b^	7.14 ^a^	1.09 ^b^	0.51 ^b^	1.86 ^ab^	2.54 ^ab^	0.71 ^b^	2.18 ^ab^	2.72 ^ab^	1.130	0.007	0.441	0.043
*Treponema_2*	0.02	3.29	3.58	0.00	0.20	2.27	0.01	1.43	8.18	1.666	0.007	0.185	0.250
*Christensenellaceae_R-7_group*	0.73	1.74	2.97	0.29	1.47	2.67	0.14	0.89	3.49	0.305	<0.001	0.384	0.125
*Ruminococcaceae_UCG-013*	0.17	2.59	3.15	0.08	0.48	2.59	0.01	0.43	3.18	0.558	<0.001	0.148	0.292
*Eubacterium_coprostanoligenes_group*	0.39	2.26	2.27	0.55	0.82	2.04	0.27	0.65	2.91	0.397	<0.001	0.331	0.076
*Ruminococcaceae_UCG-010*	0.34	1.92	2.87	0.19	1.11	2.26	0.02	0.66	2.33	0.288	<0.001	0.025	0.513
*Romboutsia*	1.50	1.37	1.97	0.49	1.05	1.46	0.71	1.55	1.44	0.253	0.007	0.027	0.373
*Turicibacter*	0.67	0.55	0.97	0.32	0.73	0.95	2.42	1.89	1.92	0.280	0.620	<0.001	0.266
*Anaerostipes*	0.99	2.33	0.16	1.18	2.72	1.99	0.47	0.01	0.01	0.568	0.104	0.001	0.176

^1^ P1 indicates phase 1, P2 indicates phase 2, P3 indicates phase 3; H = stepwise high energy and protein diet, C = stepwise standard energy and protein diet, L = stepwise low energy and protein diet. ^2^ SEM = standard error of means. ^3^ Diet = the effect of diets, Phase = the effect of phases, D × P = the interaction between diet and phase, lowercase letters are marked only when the interaction was significant. ^a,b^ values within the same row with different superscripts differ.

## Supplementary Materials

The following is available online at https://www.mdpi.com/2076-2615/9/8/560/s1, Figure S1: The relative abundance of fecal bacterial composition at the genus level.
