# Supplementary material for: Dynamic Variations in Fecal Bacterial Community and Fermentation Profile of Holstein Steers in Response to Three Stepwise Density Diets
Source: Animals (Basel). 2019 Aug 15;9(8):560. doi: 10.3390/ani9080560 (PMC6719243; doi:10.3390/ani9080560)
Supplement: Supplementary file 1 [file animals-09-00560-s001.pdf]

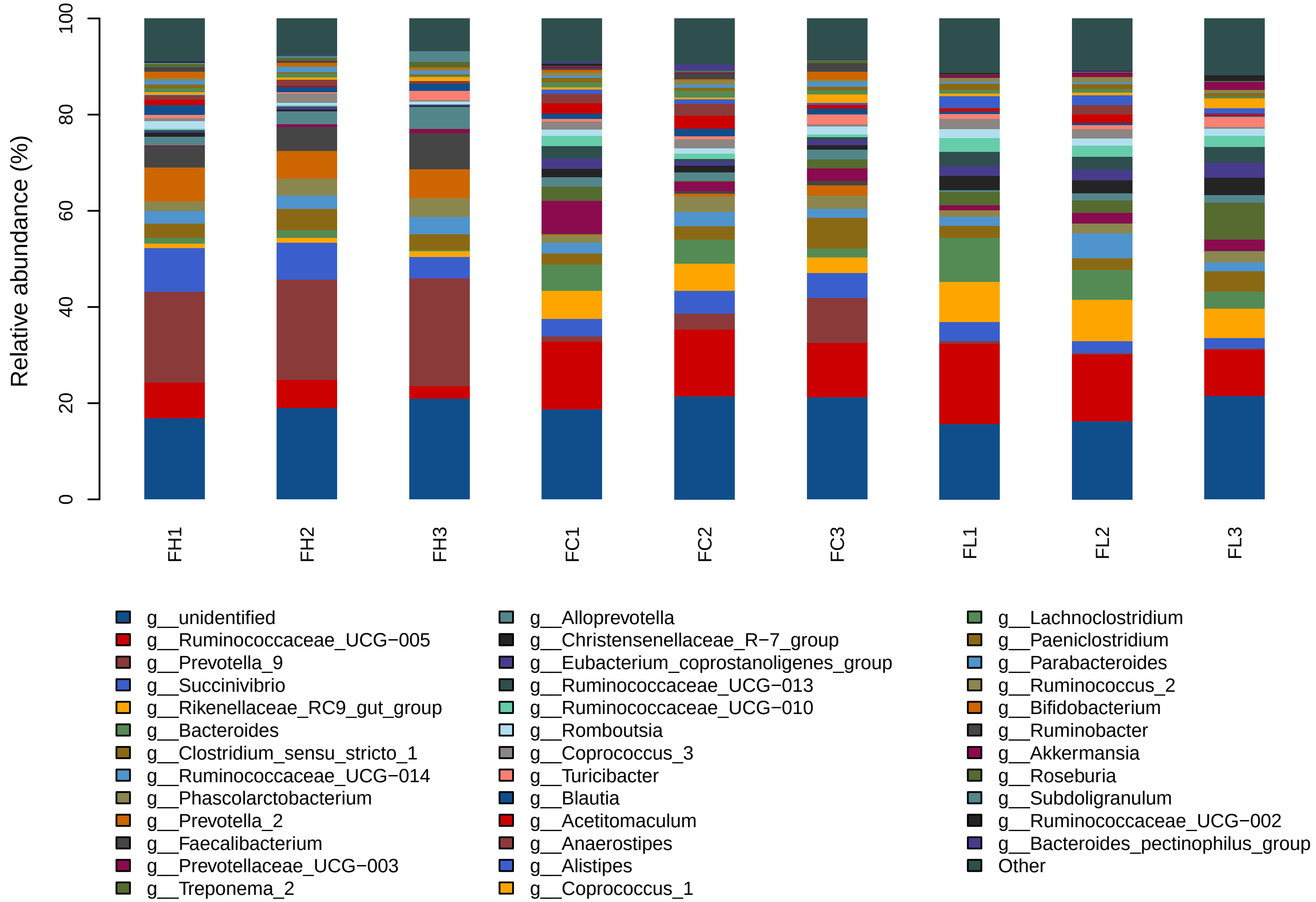

Relative abundance (%)
0
20
40
60
80
100
FH1
FH2
FH3
FC1
FC2
FC3
FL1
FL2
FL3
g__unidentified
g__Ruminococcaceae_UCG−005
g__Prevotella_9
g__Succinivibrio
g__Rikenellaceae_RC9_gut_group
g__Bacteroides
g__Clostridium_sensu_stricto_1
g__Ruminococcaceae_UCG−014
g__Phascolarctobacterium
g__Prevotella_2
g__Faecalibacterium
g__Prevotellaceae_UCG−003
g__Treponema_2
g__Alloprevotella
g__Christensenellaceae_R−7_group
g__Eubacterium_coprostanoligenes_group
g__Ruminococcaceae_UCG−013
g__Ruminococcaceae_UCG−010
g__Romboutsia
g__Coprococcus_3
g__Turicibacter
g__Blautia
g__Acetitomaculum
g__Anaerostipes
g__Alistipes
g__Coprococcus_1
g__Lachnoclostridium
g__Paeniclostridium
g__Parabacteroides
g__Ruminococcus_2
g__Bifidobacterium
g__Ruminobacter
g__Akkermansia
g__Roseburia
g__Subdoligranulum
g__Ruminococcaceae_UCG−002
g__Bacteroides_pectinophilus_group
Other
